# Computed tomography angiography in the planning of transcatheter aortic valve replacement: a step-by-step approach

**DOI:** 10.1590/0100-3984.2021.0156

**Published:** 2022

**Authors:** Írline Cordeiro de Macedo Pontes, Camila Pinto Brito de Figueiredo Guimarães, Eduardo Kaiser Ururahy Nunes Fonseca, Murilo Marques Almeida Silva, Roberto Sasdelli Neto, Walther Yoshiharu Ishikawa

**Affiliations:** 1 Hospital Israelita Albert Einstein, São Paulo, SP, Brazil.

**Keywords:** Aortic valve stenosis, Transcatheter aortic valve replacement, Computed tomography angiography, Estenose da valva aórtica, Substituição de válvula aórtica transcateter, Angiografia por tomografia computadorizada

## Abstract

Aortic valve stenosis is the most common acquired valvular heart disease. Transcatheter aortic valve implantation, also known as transcatheter aortic valve replacement (TAVR), is an important treatment option for symptomatic aortic stenosis in patients at any level of surgical risk. The role of computed tomography angiography (CTA) has expanded considerably in recent years, and it has now become the imaging method of choice for the planning of TAVR. Therefore, radiologists should understand the main aspects of this imaging modality, including the appropriate technique and protocol to acquire reliable CTA images and to create a useful radiology report. The aim of this study was to review the most important aspects of CTA for TAVR planning.

## Abstract (pt)

A estenose da válvula aórtica é a doença cardíaca valvar adquirida mais comum. A substituição transcateter da válvula aórtica (TAVR), ou implante transcateter da válvula aórtica, é uma importante opção no tratamento da estenose aórtica sintomática, independente do risco cirúrgico do paciente. O papel da angiotomografia computadorizada cresceu consideravelmente nos últimos anos e tornou-se o método de escolha no planejamento de TAVR. Assim, o radiologista deve compreender os principais aspectos dessa modalidade, incluindo técnica e protocolo, a fim de adquirir imagens confiáveis e compor um relatório para a realização do procedimento. O objetivo deste ensaio é revisar os pontos mais importantes que precisam ser avaliados na angiotomografia no planejamento de TAVR.

## INTRODUCTION

Aortic valve stenosis is the most common acquired valvular heart disease. Transcatheter aortic valve implantation, widely known as transcatheter aortic valve replacement (TAVR), has become an important treatment option for patients at any level of surgical risk^(1)^. Data from the Society of Thoracic Surgeons-American College of Cardiology Transcatheter Valve Therapy Registry demonstrate that, in the United States, the number of TAVR procedures has surpassed that of traditional surgical aortic valve replacements and continues to increase^(2)^. Computed tomography angiography (CTA) has recently become the imaging method of choice for pre-TAVR patient evaluation, planning of the procedure, and device selection^(3)^.

In this article, we review the most important points to be assessed in CTA for TAVR planning. We address the technical aspects and detail the essential information that should be included in the radiology report.

## CTA TECHNIQUE AND PROTOCOLS

The CTA protocol for TAVR planning consists of an electrocardiography (ECG)-synchronized acquisition that covers the aortic root. Non-ECG-synchronized CTA of the aorta and iliofemoral vessels is performed to evaluate the vascular access^(3)^. At our facility, CTA is performed at 120 kV, with a rotation time of 0.5 s or 0.35 s, in an acquisition incorporating radiation dose reduction strategies. The acquisition should extend from above the thoracic outlet to the proximal third of the thighs. In patients who are candidates for TAVR (i.e. patients with severe aortic stenosis), medications to reduce the heart rate are contraindicated^(4)^.

### Cardiac CTA for aortic root assessment

In CTA for assessment of the aortic root, the acquisition should account for changes in the geometry and dimensions of the aortic root during the cardiac cycle. Most required measurements are made in systole, which is when the annulus is at its largest in most patients. Measurements made in diastole are also important, in order to evaluate the morphology of the aortic valve and the coronary arteries. The CTA acquisition should encompass 30–80% of the cardiac cycle^(3)^.

### CTA of aortoiliofemoral vascularization

For the assessment of aortoiliofemoral vascularization, the CTA acquisition should include the aorta, iliac arteries, and common femoral arteries. It can also begin at the head in order to evaluate the carotid and subclavian arteries, for alternative vascular access^(4)^.

### Contrast administration

Typically, the contrast injection flow rate is 4.0–6.0 mL/s and the estimated contrast volume is 50–100 cc. The CTA acquisition can be triggered by using bolus tracking, with a region-of-interest in the descending aorta^(4)^.

### Unenhanced CT

The aortic valve calcium score is measured by the Agatston method. An aortic valve calcium score greater than 3,000 indicates an increased risk of paravalvular aortic regurgitation in the immediate post-TAVR period^(5)^.

## ANATOMY OF THE AORTIC ROOT

**Figure 1:**
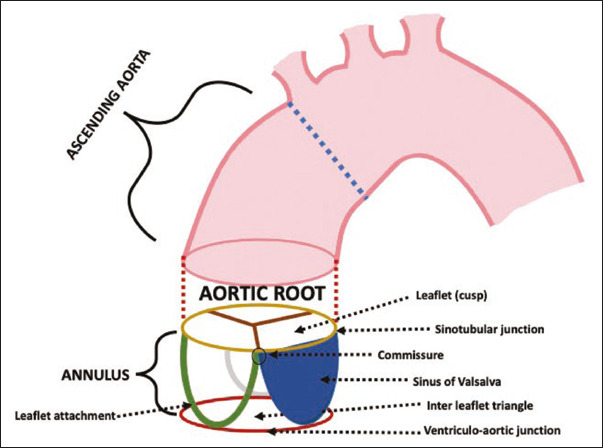
Representation of the aortic root and its components. The most widely accepted terminology describing the aortic annulus is also demonstrated.

As depicted in Figure 1, the aortic root is composed of various structures^(6)^:

- The aortic valve leaflets, which compose the aortic valve and can be divided in three parts: basal, belly, and free margin
- The sinus of Valsalva, composed of three bulges from the aortic wall, two of which give rise to the coronary arteries and are named, accordingly, the left and right sinuses, the third being referred to as the noncoronary sinus
- The interleaflet triangles, which consist of three triangular regions located under each commissure
- The sinotubular junction, which comprises the distal part of the aortic root, separating it from the ascending aorta
- The aortic annulus, a non-standardized term for what is not a true anatomical structure, typically used in order to describe a crown-like structure composed of the semilunar attachment of the three leaflets and the cylinder of connective tissue between the aortic sinuses and the left ventricular outflow tract^(7)^.

## HOW TO ASSESS AND MEASURE THE AORTIC ANNULUS

**Figure 2:**
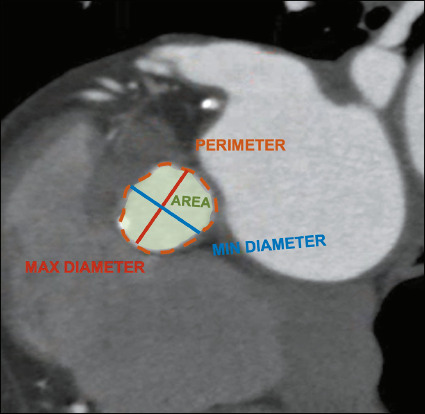
The measurements of the aortic annulus that need to be reported: the maximum diameter (in red); the minimum diameter (in blue); the area (in green); and the perimeter (in orange).

In TAVR planning, precise measurements of the aortic annulus are extremely important to achieve optimal sizing of transcatheter heart valves and to recognize patients at higher risk for coronary occlusion. For this purpose, the annulus is considered the plane in which the attachments of the three most basal cusps are aligned. The annulus is usually identified and measured manually, with multiplanar reconstruction and a contouring tool, along the bloodtissue interface^(3)^, as illustrated in Figure 2.

## IMPORTANT MEASUREMENTS AND STRUCTURAL ASSESSMENT

### Valve morphology

**Figure 3:**
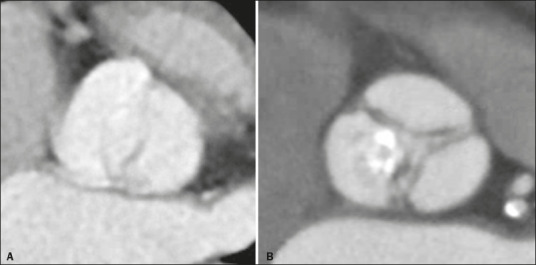
Examples of aortic valves with bicuspid and tricuspid morphologies (A and B, respectively).

Bicuspid aortic valves are associated with low TAVR success rates and higher rates of post-TAVR paravalvular regurgitation^(8)^. Bicuspid and tricuspid valve morphologies are shown in Figure 3. It is important to determine the valve opening pattern, because tricuspid valves can present a bicuspid-like pattern, as well as to describe calcifications of the leaflets and the possible insinuation of such calcifications into the subvalvular plane^(8)^.

### Landing zone calcium volume

The device landing zone encompasses the valve cusps, the aortic annulus, and the left ventricular outflow tract. The protocol that should be used in the evaluation of the degree of calcification is the same as those used in calculating the coronary artery calcium score by the Agatston method (unenhanced, ECG-synchronized CTA). Aortic valve calcification scores ≥ 3,000 in men and ≥ 1,600 in women indicate a greater likelihood of severe aortic stenosis.

### Coronary ostial height

**Figure 4:**
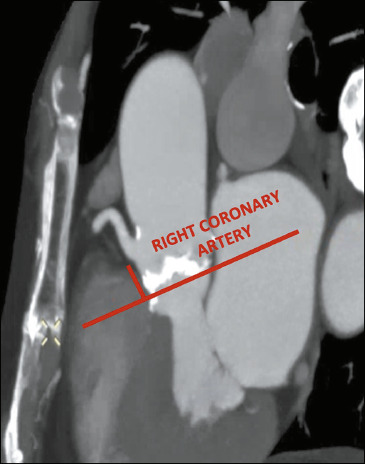
Exemplification of the measurement of the height of the right coronary ostium, from the annular plane to the lower edge of the ostium, shown in red. Low coronary ostial height (< 12 mm) increases the risk of coronary occlusion.

The height of the coronary ostium should be measured perpendicularly, from the annular plane to the lower edge of the ostium^(3)^, as shown in Figure 4.

### Sinus of Valsalva diameter

**Figure 5:**
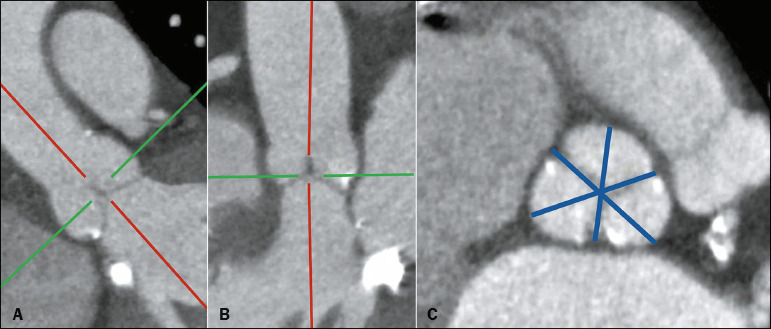
The plane in which the sinus of Valsalva should be measured (A,B). The measurements of the sinus, from cusp to commissure, are shown in blue (C). Measurements should be performed in triplicate, and, if the anatomy is symmetric, the average of the three measurements can be reported. A sinus of Valsalva with a small diameter (< 30 mm) is associated with a greater likelihood of coronary occlusion.

The diameter of the sinus of Valsalva should be measured from cusp to commissure, parallel to the annular plane^(3)^, as depicted in Figure 5.

### Sinotubular junction diameter

**Figure 6:**
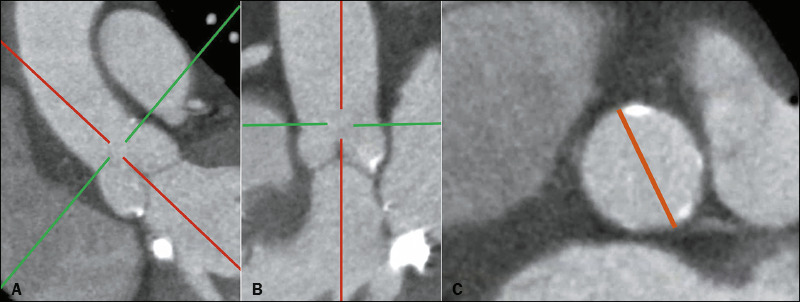
The plane in which the sinotubular junction diameter should be measured (A,B). The sinotubular junction diameter is shown in orange (C).

The diameter of the sinotubular junction should be measured in a transverse oblique plane that is aligned with the junction and usually not parallel to the annular plane^(3)^, as can be seen in Figure 6.

### Sinotubular junction height

**Figure 7:**
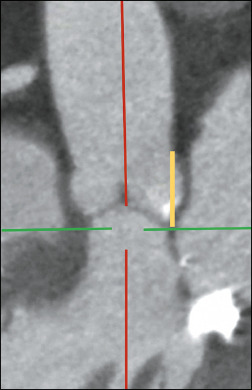
The plane in which the sinotubular junction height should be measured and the corresponding measurement (in yellow).

The height of the sinotubular junction should be measured perpendicular to the annular plane, from the annular plane to the lowest edge of the junction^(3)^, as illustrated in Figure 7.

### Ascending aorta diameter

**Figure 8:**
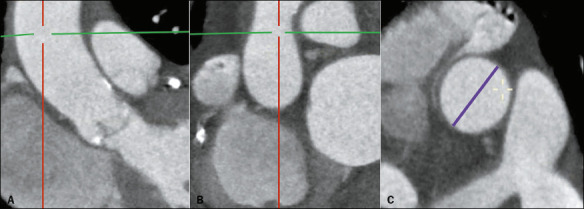
The plane in which the ascending aorta should be measured (A,B) and the corresponding measurement (in purple, in C).

The diameter of the ascending aorta should be measured in its true axial plane, at the level of its greatest width or at the level of the main pulmonary artery^(3)^, as detailed in Figure 8.

## VASCULAR ACCESS

### Aorta

The branch anatomy of the aortic arch should be described (especially if subclavian access is being considered), as should the extent of the calcifications in the aorta, as well as tortuosity, intraluminal obstruction, aneurysm, and thrombosis^(7)^.

### Iliofemoral vasculature

The iliofemoral axis is the most used route in the delivery of the most widely used transcatheter heart valves. Therefore, the evaluation of such vessels regarding size, calcifications, and tortuosity is essential to decide if transfemoral access can be achieved^(5)^. Potential contraindications to transfemoral access include peripheral artery disease, significant vessel tortuosity, severe calcification, and external sheath diameter exceeding the minimum artery diameter^(5)^. Multiple measurements should be taken in the iliofemoral vasculature, and the minimum vessel diameter on both sides should be reported. It is also essential to identify the level of femoral artery bifurcation^(3)^. The extent and distribution of calcifications should be evaluated and quantified subjectively as none, mild, moderate, or severe.

### Alternative access routes

The subclavian and carotid arteries should be examined with the same parameters described for the iliofemoral vessels. If a transapical approach is taken, CTA can provide a preprocedural anatomical detailing of the left ventricular apex^(4)^.

## OTHER IMPORTANT ASPECTS

### Cardiac findings

**Figure 9:**
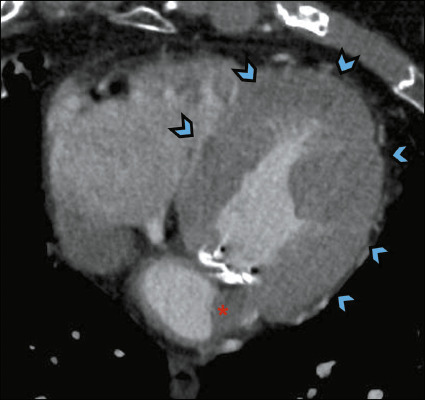
CTA for TAVR planning in a 73-year-old male patient, showing concentric hypertrophy of the left ventricular myocardium (arrowheads) and probable intracavitary thrombus in the inferior and lateral region of the left atrial cavity (asterisk).

The degree and extent of left ventricular hypertrophy should be reported, left atrial appendage occlusion should be identified, and thrombi in the wall of the left ventricle should be evaluated^(5)^, as shown in Figure 9.

### Noncardiac findings

Extracardiac pathologies and relevant incidental findings should be reported^(7)^.

## HOW TO CHOOSE THE BEST PROSTHESIS AND SIZE IT

The evaluation of the anatomical characteristics of the aortic root and accurate prosthesis sizing are crucial for the success of the intervention and require determination of the precise dimensions of the aortic annulus. Inappropriate prosthesis sizing can lead to postprocedural complications such as embolization, rupture, and paravalvular aortic regurgitation^(9)^. The manufacturer-suggested aortic root dimensions and corresponding prosthesis sizes are shown in Table 1.

**Table 1:** Manufacturer-suggested aortic root dimensions for TAVR.

Device	Prosthesis size (mm)	Aortic annulus diameter (mm)	Aortic annulus perimeter (mm)
Medtronic CoreValve	23	18–20	56.5–62.8
Evolut PRO	26	20–23	62.8–72.3
	29	23–26	72.3–81.7
	34	26–30	81.7–94.2
Edwards SAPIEN 3	20	16–19	
	23	18–22	
	26	21–25	
	29	24–28	

## THE REPORT

**Figure 10:**
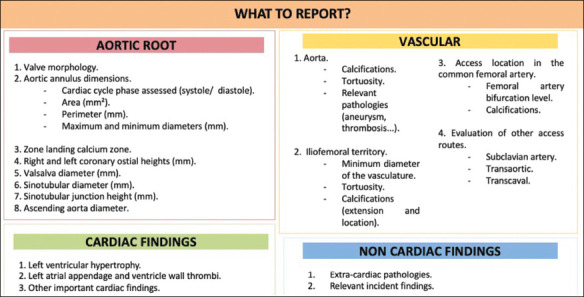
Essential information of the report in TAVR planning.

The radiology report should contain the information described in Figure 10.

## POST-TAVR COMPLICATIONS

**Figure 11:**
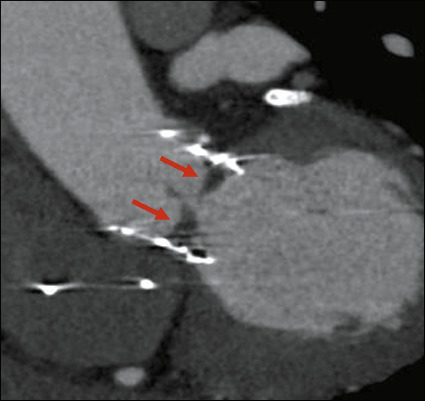
CTA of an 81-year-old male patient who had undergone TAVR one month earlier, showing a prosthetic aortic valve with hypoattenuating tissue in the valve leaflets (arrows) and partial limitation of the valve opening, suggestive of thrombosis.

The complications associated with TAVR can be divided in three groups. The first group comprises complications related to transcatheter heart valve function, expansion, and position, one of the most common complications of this type being paravalvular aortic regurgitation. Another major complication within that group is valve thrombosis, which can be visualized on CTA as crescentshaped, hypoattenuated leaflet thickening (Figure 11). The second group comprises vascular complications at the access site or along the chosen vascular route, which include dissection and arteriovenous fistula^(10)^, the most common complication in this group being pseudoaneurysm. The third group comprises all other complications, including periprocedural and postprocedural stroke^(11)^.

## CONCLUSION

Because TAVR has been widely performed, it is imperative that radiologists be familiar with the CTA protocol. They should also be able to recognize the relevant anatomical landmarks, interpret the pertinent dimensions, and include the key points in the radiology report, all of which are crucial for the success of the procedure.
